# Supplementary material for: De-novo whole genome assembly of the orange jewelweed, Impatiens capensis Meerb. (Balsaminaceae) using nanopore long-read sequencing
Source: PeerJ. 2023 Oct 23;11:e16328. doi: 10.7717/peerj.16328 (PMC10601903; doi:10.7717/peerj.16328)
Supplement: Supplemental Information 1 [file peerj-11-16328-s001.docx]

**Table S1. Annotation of repetitive elements in the *Impatiens capensis* genome**

| **Type of** | **Number of** | **Length of** | **Percent of** | |
| --- | --- | --- | --- | --- |
| **Repeat Element** | **Elements*** | **Element (bp)** | **Sequence** | |
| Retroelements | 185,784 | 348,012,960 | 47.94% |  |
| SINEs: | 0 | 0 | 0.00% |  |
| Penelope | 0 | 0 | 0.00% |  |
| LINEs: | 8,064 | 3,701,671 | 0.51% |  |
| CRE/SLACS | 0 | 0 | 0.00% |  |
| L2/CR1/Rex | 0 | 0 | 0.00% |  |
| R1/LOA/Jockey | 463 | 366,162 | 0.05% |  |
| R2/R4/NeSL | 0 | 0 | 0.00% |  |
| RTE/Bov-B | 0 | 0 | 0.00% |  |
| L1/CIN4 | 7,601 | 3,335,509 | 0.46% |  |
| LTR elements: | 177,720 | 344,311,289 | 47.43% |  |
| BEL/Pao | 0 | 0 | 0.00% |  |
| Ty1/Copia | 82,480 | 161,496,736 | 22.25% |  |
| Gypsy/DIRS1 | 90,615 | 176,167,920 | 24.27% |  |
| Retroviral | 4,625 | 6,646,633 | 0.92% |  |
| DNA transposons | 45,225 | 24,202,096 | 3.33% |  |
| hobo-Activator | 3,086 | 882,037 | 0.12% |  |
| Tc1-IS630-Pogo | 0 | 0 | 0.00% |  |
| En-Spm | 0 | 0 | 0.00% |  |
| MuDR-IS905 | 0 | 0 | 0.00% |  |
| PiggyBac | 0 | 0 | 0.00% |  |
| Tourist/Harbinger | 3,031 | 901,021 | 0.12% |  |
| Other (Mirage, P-element, Transib) | 0 | 0 | 0.00% |  |
| Rolling circles | 444 | 491,863 | 0.07% |  |
| Unclassified: | 275,087 | 141,600,499 | 19.50% |  |
| Total interspersed repeats: | 0 | 513,815,555 | 70.77% |  |
| Small RNA: | 0 | 0 | 0.00% |  |
| Satellites: | 156 | 10,463 | 0.001% |  |
| Simple repeats: | 0 | 0 | 0.00% |  |
| Low complexity: | 0 | 0 | 0.00% |  |

*Repeats fragmented by indels counted as single elements
