# Supplementary material for: De-novo whole genome assembly of the orange jewelweed, Impatiens capensis Meerb. (Balsaminaceae) using nanopore long-read sequencing
Source: PeerJ. 2023 Oct 23;11:e16328. doi: 10.7717/peerj.16328 (PMC10601903; doi:10.7717/peerj.16328)
Supplement: Supplemental Information 2 [file peerj-11-16328-s002.docx]

**Table S2a. The associated GO terms and genes grouped into categories and sub-categories for Molecular Function**

| Molecular Functions Grouped into Representative Categories and Sub-Categories  Category Sub-Category  ATP Hydrolysis Activity | Associated GO Terms (out of 1,144 MF-GO annotations)  Count Percent  190 16.61% | | Associated Genes  (out of 26,921 predicted genes; 20,046 gene annotations in MF-GO)  Count Percent Total Percent MF  1905 7.08% 9.50% | | |
| --- | --- | --- | --- | --- | --- |
|  |  |  |  |  |  |
| ATP hydrolysis activity | 62 | 5.42% | 929 | 3.45% | 4.63% |
| additional categories | 50 | 4.37% | 408 | 1.52% | 2.04% |
| hydrolase activity, hydrolyzing O-glycosyl compounds | 42 | 3.67% | 334 | 1.24% | 1.67% |
| phosphatase activity | 36 | 3.15% | 234 | 0.87% | 1.17% |
| Carbohydrate Transmembrane Transporter Activity | 82 | 7.17% | 692 | 2.57% | 3.45% |
| channel activity | 44 | 3.85% | 529 | 1.97% | 2.64% |
| additional categories | 38 | 3.32% | 163 | 0.61% | 0.81% |
| Enzyme Inhibitor Activity | 29 | 2.53% | 242 | 0.90% | 1.21% |
| enzyme inhibitor activity | 28 | 2.45% | 241 | 0.90% | 1.20% |
| additional categories | 1 | 0.09% | 1 | 0.00% | 0.00% |
| Heme Binding | 52 | 4.55% | 2200 | 8.17% | 10.97% |
| DNA binding | 2 | 0.17% | 1277 | 4.74% | 6.37% |
| additional categories | 47 | 4.11% | 385 | 1.43% | 1.92% |
| heme binding | 2 | 0.17% | 359 | 1.33% | 1.79% |
| sequence-specific DNA binding | 1 | 0.09% | 179 | 0.66% | 0.89% |
| Monooxygenase Activity | 92 | 8.04% | 684 | 2.54% | 3.41% |
| additional categories | 91 | 7.95% | 449 | 1.67% | 2.24% |
| monooxygenase activity | 1 | 0.09% | 235 | 0.87% | 1.17% |
| Oxidoreductase Activity oxidoreductase activity | 1 | 0.09% | 660 | 2.45% | 3.29% |
| Protein Binding  protein binding | 3 | 0.26% | 1746 | 6.49% | 8.71% |
|  | 1 | 0.09% | 1742 | 6.47% | 8.69% |
| additional categories | 2 | 0.17% | 4 | 0.01% | 0.02% |
| Protein Dimerization Activity | 56 | 4.90% | 858 | 3.19% | 4.28% |
| protein dimerization activity | 22 | 1.92% | 656 | 2.44% | 3.27% |
| additional categories | 34 | 2.97% | 202 | 0.75% | 1.01% |
| Protein Kinase Activity | 359 | 31.38% | 3536 | 13.13% | 17.64% |
| protein kinase activity | 33 | 2.88% | 1229 | 4.57% | 6.13% |
| additional categories | 170 | 14.86% | 631 | 2.34% | 3.15% |
| serine-type endopeptidase activity | 24 | 2.10% | 360 | 1.34% | 1.80% |
| UDP-glycosyltransferase activity | 45 | 3.93% | 349 | 1.30% | 1.74% |
| glycosyltransferase activity | 1 | 0.09% | 295 | 1.10% | 1.47% |
| acyltransferase activity, transferring groups other than amino-acyl groups | 42 | 3.67% | 236 | 0.88% | 1.18% |
| methyltransferase activity | 38 | 3.32% | 235 | 0.87% | 1.17% |
| ubiquitin-protein transferase activity | 6 | 0.52% | 201 | 0.75% | 1.00% |
| Structural Constituent of Ribosome structural constituent of ribosome | 10 | 0.87% | 299 | 1.11% | 1.49% |
| Transferase Activity  transferase activity | 2 | 0.17% | 1574 | 5.85% | 7.85% |
|  | 1 | 0.09% | 961 | 3.57% | 4.79% |
| hydrolase activity | 1 | 0.09% | 613 | 2.28% | 3.06% |
| Zinc Ion Binding | 36 | 3.15% | 4318 | 16.04% | 21.54% |
| GTP binding | 1 | 0.09% | 1899 | 7.05% | 9.47% |
| zinc ion binding | 8 | 0.70% | 1283 | 4.77% | 6.40% |
| pyridoxal phosphate binding | 3 | 0.26% | 541 | 2.01% | 2.70% |
| nucleic acid binding | 1 | 0.09% | 385 | 1.43% | 1.92% |
| calcium ion binding | 1 | 0.09% | 210 | 0.78% | 1.05% |
| Additional Categories | 232 | 20.28% | 1332 | 4.95% | 6.64% |
