# Supplementary material for: De-novo whole genome assembly of the orange jewelweed, Impatiens capensis Meerb. (Balsaminaceae) using nanopore long-read sequencing
Source: PeerJ. 2023 Oct 23;11:e16328. doi: 10.7717/peerj.16328 (PMC10601903; doi:10.7717/peerj.16328)
Supplement: Supplemental Information 3 [file peerj-11-16328-s003.docx]

**Table S2b. The associated GO terms and genes grouped into categories and sub-categories for Biological Process**

| Biological Processes Grouped into Representative Categories and Sub-Categories  Category Sub-Category  Carbohydrate Metabolic Process | | Associated GO Terms (out of 1,599 BP-GO annotations)  Count Percent  1 0.06% | | Associated Genes  (out of 26,921 predicted genes; 12,696 genes in BP-GO)  Count Percent Total Percent BP | | |
| --- | --- | --- | --- | --- | --- | --- |
|  | |  | | 368 1.37% 2.90% | | |
| *Cell Redox Homeostasis* | | *24* | *1.50%* | *110* | *0.41% 0.38%* | *0.87% 0.81%* |
| cell redox homeostasis | | 23 | 1.44% | 103 | 0.38% | 0.81% |
| additional categories | | 1 | 0.06% | 7 | 0.03% | 0.06% |
| *Cell Wall Organization* | | *122* | *7.63%* | *669* | *2.49%* | *5.27%* |
| cell wall organization | | 59 | 3.69% | 440 | 1.63% | 3.47% |
| additional categories | | 63 | 3.94% | 229 | 0.85% | 1.80% |
| *Defense Response* | | *171* | *10.69%* | *1367* | *5.08%* | *10.77%* |
| defense response | | 23 | 1.44% | 418 | 1.55% | 3.29% |
| cellular oxidant detoxification | | 28 | 1.75% | 365 | 1.36% | 2.87% |
| intracellular signal transduction | | 7 | 0.44% | 221 | 0.82% | 1.74% |
| response to light stimulus | | 56 | 3.50% | 185 | 0.69% | 1.46% |
| additional categories | | 57 | 3.56% | 178 | 0.66% | 1.40% |
| *Lipid Metabolic Process* lipid metabolic process | | *1* | *0.06%* | *124* | *0.46%* | *0.98%* |
| *Metabolic Process* metabolic process | | *1* | *0.06%* | *162* | *0.60%* | *1.28%* |
| *Methylation* methylation | | *1* | *0.06%* | *110* | *0.41%* | *0.87%* |
| *Microtubule-based Movement*  *microtubule-based movement* | | *15* | *0.94%* | *114* | *0.42%* | *0.90%* |
|  | microtubule-based movement | 13 | 0.81% | 112 | 0.42% | 0.88% |
| additional categories | | 2 | 0.13% | 2 | 0.01% | 0.02% |
| *Protein Phosphorylation* | | *680* | *42.53%* | *5216* | *19.38%* | *41.08%* |
| protein phosphorylation | | 106 | 6.63% | 1886 | 7.01% | 14.86% |
| mRNA splicing, via spliceosome | | 65 | 4.07% | 573 | 2.13% | 4.51% |
| ubiquitin-dependent protein catabolic process | | 62 | 3.88% | 406 | 1.51% | 3.20% |
| proteolysis | | 1 | 0.06% | 324 | 1.20% | 2.55% |
| phosphatidylinositol phosphate biosynthetic process | | 69 | 4.32% | 261 | 0.97% | 2.06% |
| cellulose biosynthetic process | | 28 | 1.75% | 228 | 0.85% | 1.80% |
| aromatic amino acid family biosynthetic process | | 53 | 3.31% | 189 | 0.70% | 1.49% |
| DNA-templated transcription initiation | | 3 | 0.19% | 165 | 0.61% | 1.30% |
| peptidyl-serine phosphorylation | | 36 | 2.25% | 105 | 0.39% | 0.83% |
| additional categories | | 257 | 16.07% | 1079 | 4.01% | 8.50% |
| *Protein Transport* | | *200* | *12.51%* | *1855* | *6.89%* | *14.61%* |
| monoatomic ion transport | | 2 | 0.13% | 616 | 2.29% | 4.85% |
| protein transport | | 95 | 5.94% | 558 | 2.07% | 4.40% |
| monoatomic ion transmembrane transport | | 29 | 1.81% | 270 | 1.00% | 2.13% |
| vesicle-mediated transport | | 1 | 0.06% | 121 | 0.45% | 0.95% |
| exocytosis | | 16 | 1.00% | 102 | 0.38% | 0.80% |
| additional categories | | 57 | 3.56% | 188 | 0.70% | 1.48% |
| *Regulation of Transcription by RNA polymerase II* | | *209* | *13.07%* | *1639* | *6.09%* | *12.91%* |
| regulation of transcription by RNA polymerase II | | 4 | 0.25% | 876 | 3.25% | 6.90% |
| regulation of catalytic activity | | 32 | 2.00% | 180 | 0.67% | 1.42% |
| positive regulation of DNA-templated transcription | | 36 | 2.25% | 148 | 0.55% | 1.17% |
| additional categories | | 137 | 8.57% | 435 | 1.62% | 3.43% |
| *Root Development* | | *105* | *6.57%* | *278* | *1.03%* | *2.19%* |
| root development | | 67 | 4.19% | 200 | 0.74% | 1.58% |
| additional categories | | 38 | 2.38% | 78 | 0.29% | 0.61% |
| *Additional Categories* | | *69* | *4.32%* | *684* | *2.54%* | *5.39%* |
