# Supplementary material for: De-novo whole genome assembly of the orange jewelweed, Impatiens capensis Meerb. (Balsaminaceae) using nanopore long-read sequencing
Source: PeerJ. 2023 Oct 23;11:e16328. doi: 10.7717/peerj.16328 (PMC10601903; doi:10.7717/peerj.16328)
Supplement: Supplemental Information 4 [file peerj-11-16328-s004.docx]

**Table S2c. The associated GO terms and genes grouped into categories and sub-categories for Cellular Component**

| *Cellular Components Grouped into Representative Categories and Sub-Categories*  *Category Sub-Category* | *Associated GO Terms (out of 424 CC-GO annotations)*  *Count Percent*  *2 0.47%* | | *Associated Genes*  *(out of 26,921 predicted genes; 7,851 genes in CC-GO)*  *Count Percent Total Percent CC* | | |
| --- | --- | --- | --- | --- | --- |
| *Apoplast* apoplast | *2 0.47%* | | *83 0.31% 1.06%* | | |
| *Cell Wall* | *113* | *26.65%*  *0.24%* | *1006* | *3.74%*  *1.32%* | *12.81%*  *4.53%* |
| plasma membrane | 1 | 0.24% | 356 | 1.32% | 4.53% |
| cell wall | 4 | 0.94% | 136 | 0.51% | 1.73% |
| membrane coat | 24 | 5.66% | 87 | 0.32% | 1.11% |
| additional categories | 84 | 19.81% | 427 | 1.59% | 5.44% |
| *Cytoplasm* | *274* | *64.62%* | *4122* | *15.31%* | *52.50%* |
| nucleus | 2 | 0.47% | 1202 | 4.46% | 15.31% |
| cytoplasm | 1 | 0.24% | 528 | 1.96% | 6.73% |
| ribosome | 41 | 9.67% | 520 | 1.93% | 6.62% |
| Golgi apparatus | 16 | 3.77% | 336 | 1.25% | 4.28% |
| chloroplast | 23 | 5.42% | 276 | 1.03% | 3.52% |
| cytosol | 1 | 0.24% | 186 | 0.69% | 2.37% |
| nucleosome | 37 | 8.73% | 172 | 0.64% | 2.19% |
| spliceosomal complex | 48 | 11.32% | 168 | 0.62% | 2.14% |
| mitochondrion | 1 | 0.24% | 127 | 0.47% | 1.62% |
| cytosolic large ribosomal subunit | 9 | 2.12% | 116 | 0.43% | 1.48% |
| mitochondrial inner membrane | 19 | 4.48% | 91 | 0.34% | 1.16% |
| additional categories | 76 | 17.92% | 400 | 1.49% | 5.09% |
| *Extracellular Region* extracellular region | *1* | *0.24%* | *184* | *0.68%* | *2.34%* |
| *Membrane* membrane | *1* | *0.24%* | *2269* | *8.43%* | *28.90%* |
| *Additional Categories* | *33* | *7.78%* | *187* | *0.69%* | *2.38%* |
