# Supplementary figures and images for: De-novo whole genome assembly of the orange jewelweed, Impatiens capensis Meerb. (Balsaminaceae) using nanopore long-read sequencing
Source: PeerJ. 2023 Oct 23;11:e16328. doi: 10.7717/peerj.16328 (PMC10601903; doi:10.7717/peerj.16328)

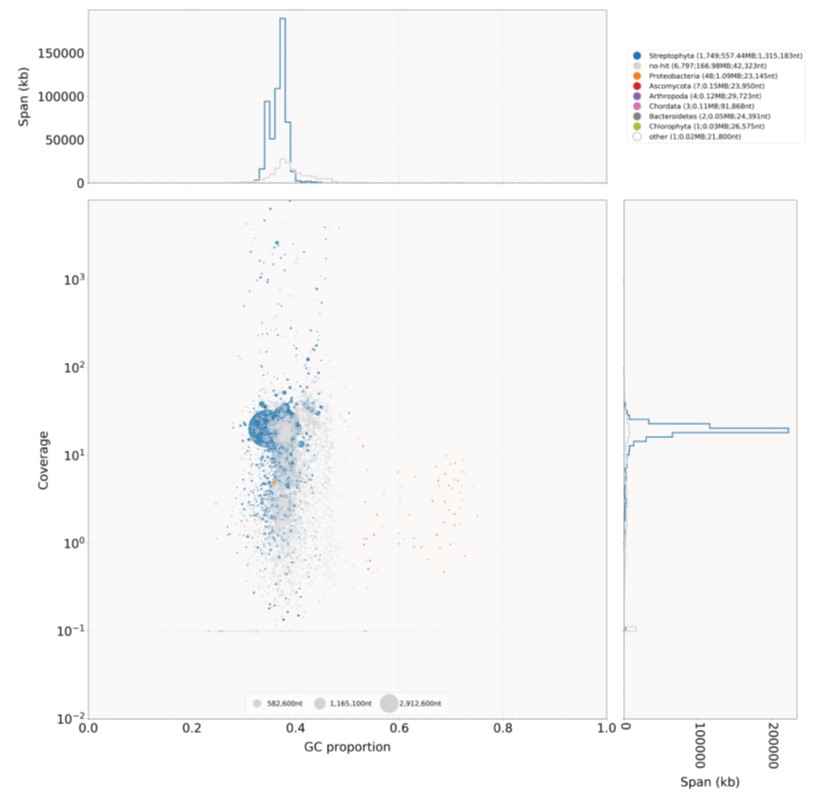

Supplement: Supplemental Information 5 — Sequences in the assembly are depicted as circles, with the diameters scaled proportional to the sequence length and colored by taxonomic annotation [file peerj-11-16328-s005.jpg]

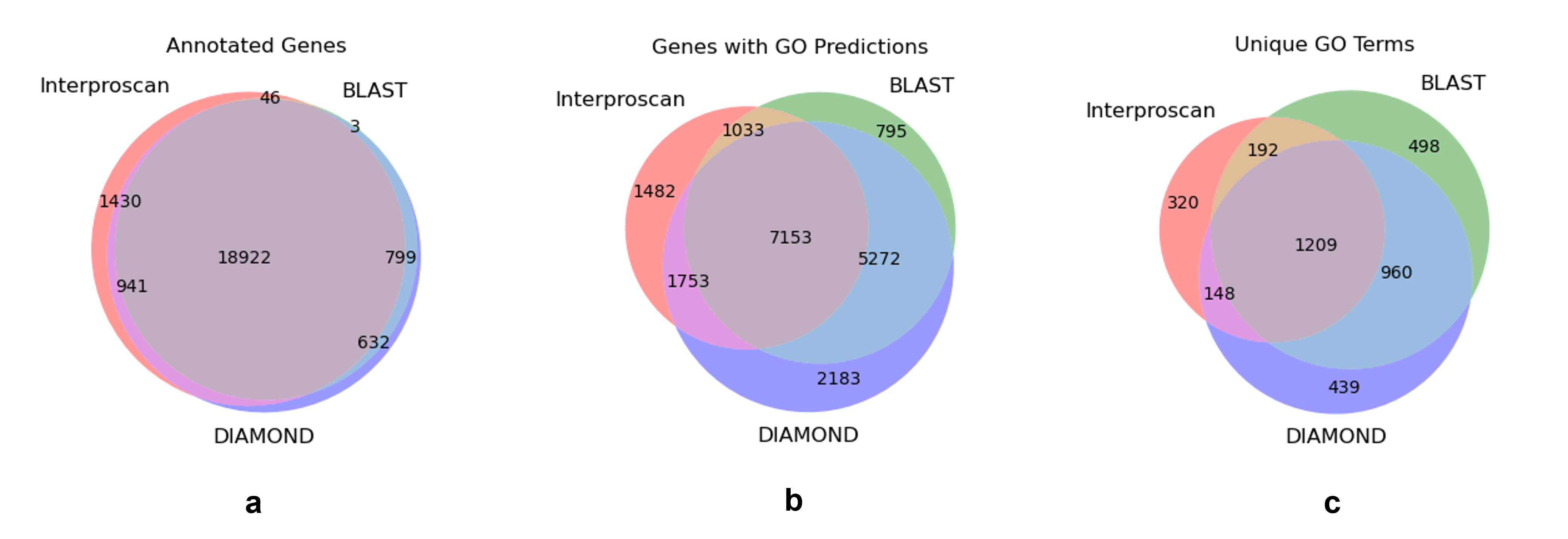

Supplement: Supplemental Information 7 [file peerj-11-16328-s007.png]

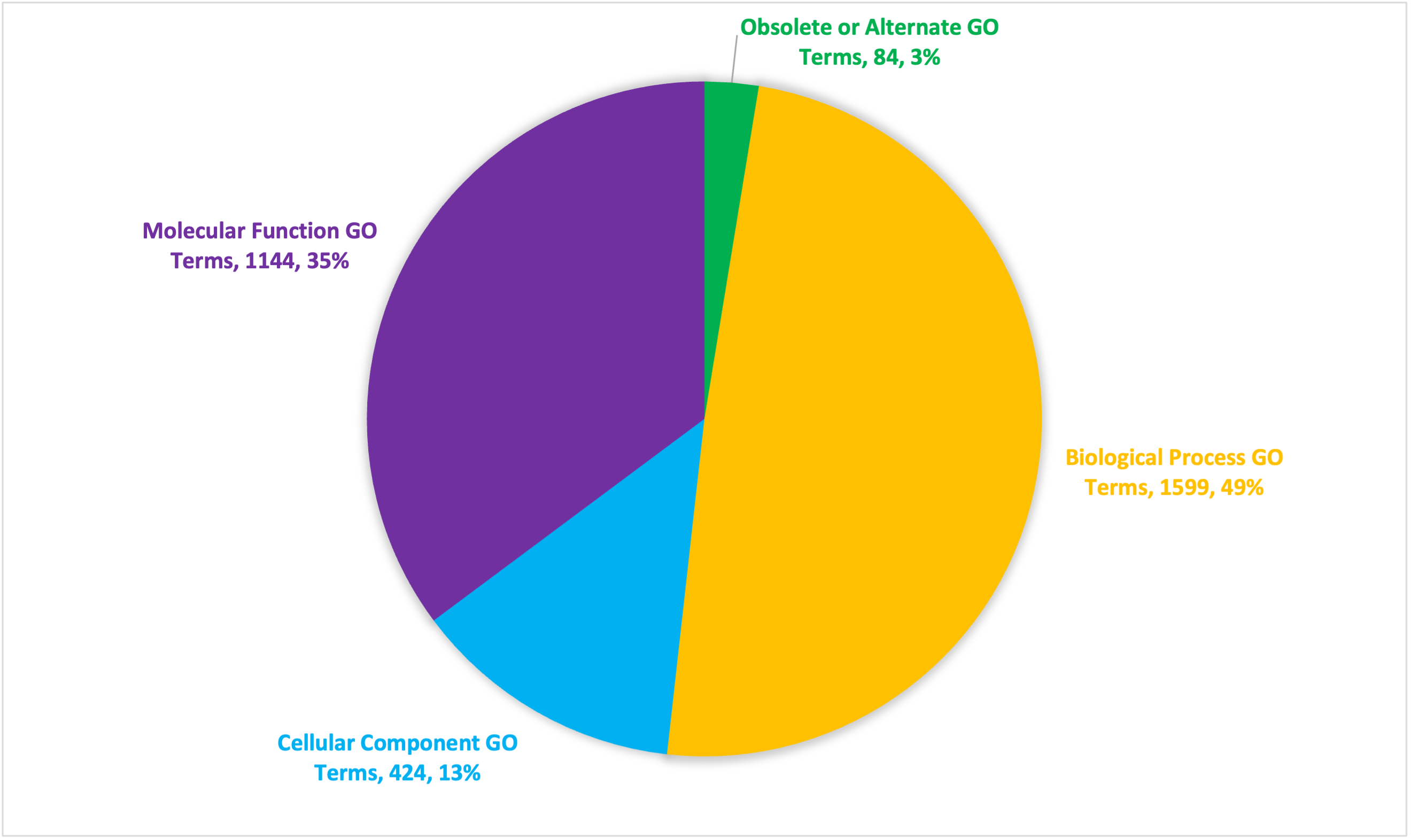

Supplement: Supplemental Information 8 [file peerj-11-16328-s008.png]
